# An Evaluation of the Sealing Ability of MTA and Resilon: A Bacterial Leakage Study

**Published:** 2007-07-05

**Authors:** Zahed Mohammadi, Abbasali Khademi

**Affiliations:** 1 *Department of Endodontics, Dental School, Shaheed Sadoughi University of Medical Sciences, Yazd, Iran*; 2 *Department of Endodontics, Dental School, Isfahan University of Medical Sciences, Isfahan, Iran*

**Keywords:** Coronal Leakage, *Enterococcus Faecalis*, MTA, Root Canal Obturation, Resilon

## Abstract

**INTRODUCTION:** The purpose of this study was to evaluate the sealing ability of gray-colored mineral trioxide aggregate (GMTA), white-colored MTA (WMTA), and Resilon as root filling materials.

**MATERIALS AND METHODS:** Sixty-six human maxillary central incisors were used in the present study. In the group A, 20 teeth were filled with GMTA. In the group B, 20 teeth were filled with WMTA and in the group C, 20 teeth were filled with Resilon/Epiphany. Three teeth were used as positive (obturated using the single gutta-percha cone technique without sealer) and three were used as negative (obturated with gutta-percha and AH-26 sealer, coated with two layers of nail varnish) controls. A bacterial leakage model utilizing *Enterococcus faecalis *was used for evaluation. Leakage was noted when turbidity was observed.

**RESULTS:** Controls behaved as expected. In the group A (GMTA) three samples, in the group B (WMTA) four samples, and in the group C (Resilon/Epiphany), two samples were leaked. There was no statistically significant difference between GMTA and WMTA or GMTA and Resilon.

**CONCLUSION:** In conclusion, sealing ability of GMTA and WMTA was similar to Resilon as orthograde root filling materials.

## INTRODUCTION

Microorganisms play an essential role in pulpal and periapical diseases (1-3). Therefore, the purpose of endodontic treatment is to eliminate microorganisms from the root canal system and to prevent recontamination by creating a seal barrier between the oral microflora, root canal system, and periapical tissue. In reality, creating a fluid-tight apical, lateral, and coronal seal is necessary to prevent recontamination and long-term clinical success (4). Several root-filling materials and techniques have been developed with the purpose of obtaining a tight root canal seal. Ideally, the root canal filling should be a complete, homogenous mass that fills the prepared canal completely.

A new material, Resilon™ (Epiphany™, Pentron Clinical Technologies, Wallingford, CT, USA; RealSeal™, SybronEndo, Orange, CA, USA), has been developed to replace gutta-percha and traditional sealers for root canal obturation. It is a thermoplastic synthetic polymer-based root-canal filling material. In addition, Epiphany is a dual curable dental resin composite. According to the manufacturer, Resilon™ sealer bonds to root canal filling material and to dentin.

Mineral trioxide aggregate (MTA) has a variety of potential uses, including as a root canal obturating material. Studies have demonstrated encouraging regeneration of periradicular tissues, such as periodontal ligament, bone, and cementum, when MTA was used in endodontic procedures (5-7). There are also several reports of its superior biocompatibility with periodontal tissues (8-9), excellent sealing ability in the presence of moisture (10-11), and appropriate mechanical properties as apical sealing material (8). These encouraging outcomes from in-vivo and in-vitro studies have prompted many clinicians to consider the use of MTA as a root end filling material and as a material suitable for repairing perforations and performing apexification (5-7).

MTA has been used for apexification of immature roots instead of Ca(OH)2 because of its facilitation of normal periradicular architecture by inducing hard tissue barriers (5). MTA has also presented promising outcomes when used for the repair of lateral and furcation perforations. Formation of cementum surrounding MTA was observed, even after extrusion of MTA into a furcation (12). On the basis of these findings, MTA may be an appropriate material for sealing of immature root canals as well as mature root canals with open apices, which may impose technical challenges in obtaining adequate obturation because of apical perforation, over-instrumentation, resorption, or former surgical treatment. Successful prognosis from conservative treatment with MTA for such difficult cases without surgical treatment would be of great benefit for patients. The purpose of this study was to compare the sealing ability of GMTA, WMTA, and Resilon as orthograde root filling materials.

## MATERIALS AND METHODS

Sixty-six extracted mature human maxillary central incisors with large canals were used for this study. After preparation of coronal access, working length was determined by placing #30 K-file through the canal space until it could be visualized exiting the apical foramen. Working length was determined by subtracting 1 mm from the canal length. The coronal two thirds of the canals were prepared sequentially with size 2 and 3 Gates-Glidden burs (Dentsply, Maillefer). The apical third of the canal was instrumented up to size 100 to simulate open-apex teeth. Irrigation was carried out using 5 mL of a 5.25% NaOCl solution between files. After preparation, the canals were irrigated with 5 ml 17% EDTA for three minutes to remove the smear layer, followed by 5 ml 5.25% NaOCl. The final irrigation was done with 5 ml distilled water. The canals were then dried with sterile paper points.

Teeth were divided into three experimental groups of 20 teeth each and two control groups of 3 teeth each. In groups A and B, roots were filled with GMTA (ProRoot MTA, Tulsa Dental, Tulsa, OK, USA) and WMTA (ProRoot MTA, Tulsa Dental, Tulsa, OK, USA), respectively. In both two groups, a thick mix of MTA was prepared and applied to the apical portion of the canal using plugger and were compacted using the butt end of paper points. In the group C, teeth were filled with Resilon™ (Epiphany™, Pentron Clinical Technologies, Wallingford, CT, USA; RealSeal™, Sybron-Endo, Orange, CA, USA). Three roots were obturated using the single gutta-percha cone (#100) technique without sealer and served as positive controls. Three roots were obturated with gutta-percha and AH-26 sealer (Dentsply, De Trey, Konstanz, Germany), coated with two layers of nail varnish (entire root plus apical foramen) and served as negative controls. Each tooth was radiographed to confirm the length and density of the root canal obturation. Each tooth was then individually sealed in a plastic vial in 100% humidity and placed in an incubator at 37°C for 48h to allow the obturation materials to set. Next, the teeth were removed from the vials, dried and then coated with two layers of nail varnish leaving the apical 3-mm of the roots and the coronal access cavities exposed.

Glass tubes equipped with microcaps were used to suspend the prepared teeth in Brain Heart Infusion (BHI) broth. A hole was made through the centre of each cap and the tooth was placed into the hole to the cementoenamel junction. The gap between the tooth and the hole was filled with sticky wax. The completed apparatus was then sterilized with ethylene dioxide. A 24-h broth culture of *Enterococcus* *faecalis* was placed into the pulp chamber of the tooth was suspended in sterile BHI broth to a level sufficient to cover the apical 3mm of the root tip. Tubes were incubated at 37°C until the BHI broth became turbid, indicating bacterial growth. Fresh 24-h cultures of *E. faecalis* were added every two days throughout the study. Turbidity of the broth was recorded daily for a total period of 90 days. Fisher exact test was used to show any significant differences. Significance was established at *p*< 0.05.

## RESULTS

**Figure1:**
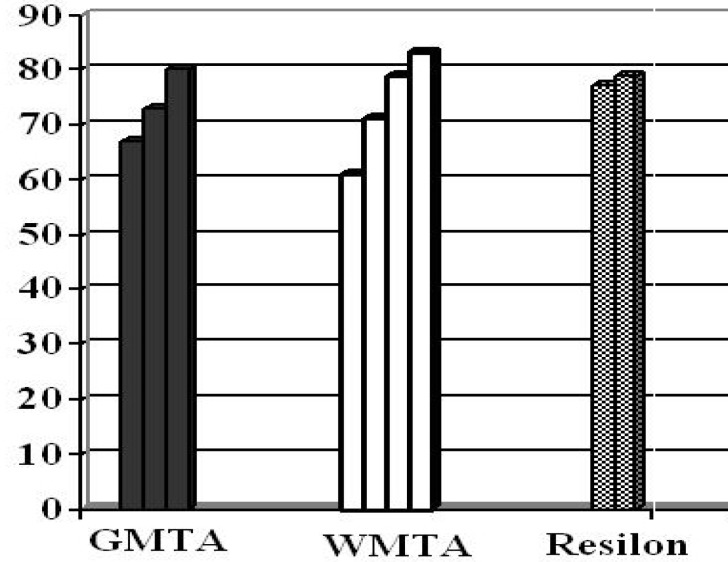
Material leakage time (days)

At the end of 90 days, in both groups A and B, leakage was observed in three and four samples, respectively. In the group C, leakage was observed in two samples. All roots in the positive control group showed broth turbidity within 48h. Roots in the negative control group did not show broth turbidity during the entire monitoring period. Statistically, there was no significant difference in leakage between GMTA and WMTA or between GMTA and Resilon (*p *>0.05) (Figure 1).

## DISCUSSION

Three-dimensional sealing of the root canal is one of the main goals of endodontic treatment and is essential for preventing apical and coronal leakage in the root-canal system.

Several test methods have been described to evaluate sealing quality of obturated root canals. In the present study, a bacterial leakage model was used because it is more reliable than dye penetration and fluid filtration methods (14). Controls behaved as expected which confirms the method. Al-Hezaimi *et al.* (14) assessed the sealing ability of GMTA and WMTA for a total period of 42 days and found that GMTA, as well as WMTA, had better sealing ability than gutta-percha and Kerr Canal Sealer EWT. Their findings regarding the MTA cements are in accordance to the results of the present study. Furthermore, the results of the present study differ from those of Vizgirda *et al.* (15), who reported that the apical seal produced by laterally- condensed gutta-percha and sealer was superior to that produced by MTA. The difference could be attributed to the some variables. In the present study, leakage was measured by bacterial penetration as opposed to dye penetration. Further, human teeth were used here instead of bovine teeth. In the Epiphany root obturation system, Resilon™ sealer’s attachment to root canal walls and to the Resilon™ filling core material appears to be superior. The better sealing ability of Resilon™ System may be attributed to the ‘monoblock’ provided by the adhesion of the filling material to the sealer, which also adheres and penetrates into the dentin walls of the root-canal system. Shipper *et al.* (13) compared bacterial leakage using *Streptococcus mutans* and *E. faecalis* through gutta-percha and Resilon during a 30-day period. Resilon showed minimal leakage, which was significantly less than gutta-percha, in which approximately 80% of specimens leaked. In another study, Bodrumlu and Tunga (16) compared the apical sealing ability of teeth filled with gutta-percha/AH-26 sealer to that of Resilon. Results revealed that the teeth filled with gutta-percha /AH -26 displayed the most apical leakage and the least apical leakage was shown with Resilon.

In the present study, the difference between the two formulations of MTA was not statistically significant. Ferris and Baumgartner (17) compared the ability of GMTA and WMTA to seal furcation perforations in vitro and found no difference between the two preparations. Parirokh *et al*. (18) compared GMTA and WMTA as pulp capping agents in dog’s teeth and found that calcified bridge could be seen 1 week after treatment with both types of MTA, with no significant differences. In another study, De-Deus *et al.* (19) compared the ability of Portland cement and MTA to prevent coronal leakage through repaired furcal perforations in molar teeth and found that there was no statistically significant difference between the two groups.

Clinical support for the use of MTA as an obturating material, however, was presented in some case reports. In a case report, O’Sullivan and Hartwell (20) used MTA as the obturating material for the root canal system of a retained primary second molar. At the 4-month follow-up, the patient was asymptomatic, clinical findings were within normal limits, and there was evidence of radiographic healing. In another case report, Hayashi *et al.* (21) used MTA for obturation of the root canal system of two mature mandibular central incisors with apical periodontitis. A 2-year follow-up radiographic examination demonstrated the dramatic regeneration of periradicular tissue.

Considering the results of the present study and other above – mentioned studies, it seems that GMTA and WMTA may be equally useful for a variety of clinical applications.

Extrapolation of the result of this in-vitro study to clinical situation must be performed with caution. Post space preparation is often required immediately following root canal obturation. In addition, retrieving of the set MTA from the root canal is difficult if nonsurgical retreatment is indicated. Therefore, orthograde root canal filling with MTA should be limited to selected cases such as one – visit apexification and situations where future nonsurgical retreatment is nonfeasible or may not render better tooth prognosis.

## CONCLUSION

In conclusion, within the limitations of the present study the coronal seal produced by MTA preparations was equal to that produced by Resilon.
